# Impact of disease location and laterality on hemodynamic response following pulmonary thromboendarterectomy for chronic thromboembolic pulmonary hypertension^☆^

**DOI:** 10.1016/j.jhlto.2025.100314

**Published:** 2025-06-14

**Authors:** Bryan O. Pérez Martínez, Gabriella V. Rubick, Avi Toiv, Sidney Perkins, Jorge Vinales, Victor M. Moles, Vallerie V. McLaughlin, Thomas M. Cascino, Bryan Kelly, Gillian Grafton, Rana Awdish, Jonathan W. Haft, Vikas Aggarwal

**Affiliations:** a Department of Internal Medicine, University of Michigan, Ann Arbor, MI; b Division of Cardiology, Department of Internal Medicine, University of Connecticut Health, Farmington, CT; c Department of Internal Medicine, Henry Ford Health System, Detroit, MI; d University of Michigan Medical School, Ann Arbor, MI; e Division of Cardiology (Frankel Cardiovascular Center), Department of Internal Medicine, University of Michigan, Ann Arbor, MI; f Division of Pulmonary and Critical Care Medicine, Department of Internal Medicine, Henry Ford Health System, Detroit, MI; g Department of Osteopathic Medical Specialties, MIchigan State University College of Osteopathic Medicine, East Lansing, MI; h Division of Cardiology, Department of Internal Medicine, Henry Ford Health System, Detroit, MI; i Michigan State College of Human Medicine, Lansing, MI; j Department of Cardiac Surgery, University of Michigan, Ann Arbor, MI

**Keywords:** CTEPH, Pulmonary hypertension, PTE, PVR, Obstruction, Angiogram

## Abstract

**Background:**

In patients with chronic thromboembolic pulmonary hypertension (CTEPH) undergoing pulmonary thromboendarterectomy (PTE), obstructive disease burden predicts positive hemodynamic responsiveness. However, the effect of disease location (upper, middle, or lower lobes) and lung laterality (right or left) has not been studied.

**Objectives:**

Examine the effect of obstructive disease location and laterality on hemodynamic response following PTE.

**Methods:**

This analysis is a retrospective cohort study of 56 consecutive patients diagnosed with CTEPH who underwent PTE at the University of Michigan Hospital between August 2019 and July 2022. Disease burden, location, and laterality were assessed on invasive pulmonary angiography (IPA), and lobar segments were assigned a score based on these features and correlated with an absolute change in pulmonary vascular resistance (PVR) following PTE. The relationship between disease burden and hemodynamic responsiveness was modeled using linear regressions with *R*^2^ reported as a measure of correlation.

**Results:**

Most patients were World Health Organization (WHO) class III or IV (*n* = 47; 83.9%) and had a history of acute pulmonary embolism (*n* = 51; 91.1%). A modest correlation between patients’ overall disease burden and absolute change in PVR was noted, with the strongest contributions from the right lower lobe (RLL), right middle lobe (RML), and left lower lobe (LLL) (*R*^2^ = 0.16, 0.10, and 0.03, respectively).

**Conclusion:**

Disease location in the RLL, RML, and LLL may predict hemodynamic improvement in patients with CTEPH undergoing PTE.

## Background

Traditionally, pulmonary thromboendarterectomy (PTE) has been the gold standard therapeutic procedure for chronic thromboembolic pulmonary hypertension (CTEPH) in patients who are believed to have surgically accessible disease, as determined by a high-volume center.^1^, ^2^, ^3^ This assessment involves multidisciplinary discussion, and a critical component of that assessment is anatomic disease accessibility analysis. To determine if a patient's condition can be treated with surgery, invasive pulmonary angiography (IPA) and/or cross-sectional computed tomography angiography are used to assess anatomic clot burden.^4^, ^5^

Historically, the Jamieson classification for disease location was proposed using the surgical thrombi specimens obtained during PTE, wherein all patients undergoing PTE were subdivided into levels 1-4 postoperatively.^6^ More recently, a new classification for subdividing disease level in CTEPH on IPA has been proposed, wherein level 1 is defined as evidence of chronic thrombus in the main pulmonary artery branches.^7^ Under this same classification, level 2 has been defined as disease in the lobar artery branches, level 3 is disease at the level of the proximal segmental pulmonary artery branches, and level 4 is defined as evidence of disease on the distal and sub-segmental artery branches.^7^

At many CTEPH centers, available imaging is used to guide surgical decisions, often based on expert opinion. However, limited data exists regarding which anatomic features can reliably predict a positive hemodynamic response after PTE surgery. The effect of each lung lobe on hemodynamic response following surgery remains unclear. To address this issue, we analyzed disease level, location, and laterality on IPA in a series of patients who underwent PTE over 3 years. Our objective is to enhance our ability to anticipate the hemodynamic response to PTE by evaluating the location and laterality of disease burden pre-operatively.

## Methods

We performed a single-center retrospective cohort study of 56 consecutive adult patients undergoing PTE between January 1st, 2019, and March 31st, 2023. This study was approved by the University of Michigan Institutional Review Board (HUM 00208936).

### Inclusion criteria

Fifty-six (56) consecutive patients met our inclusion criteria, which required that they be over 18 years old, have a confirmed diagnosis of CTEPH, have a baseline right heart catheterization (RHC) with IPA performed in accordance with our center protocol, undergo PTE at the University of Michigan Hospital, and undergo post-PTE RHC follow-up afterward. Exclusion criteria included patients with right or left main pulmonary artery total occlusion.

### Data collection

De-identified data was collected using REDCap 13.1.30 and exported to Microsoft Excel 2023. Two authors (BPM, AT) were responsible for initial data entry, and a third author (GVR) conducted random data reviews for accuracy. Data collected includes pre-operative and diagnostic characteristics, operative information, and post-surgical follow-up information. All participants had pre- and post-operative RHC, for which the hemodynamics were collected. One author (VA) performed and interpreted all IPAs included in this analysis; given that this was done prior to surgery, every interpretation was blinded to the post-PTE hemodynamic changes.

### Outcomes

The primary outcome was the absolute change in pulmonary vascular resistance (PVR) following PTE.

### Modeling the effect of disease burden on hemodynamics

IPAs were performed and interpreted in accordance with our previously published protocol.^8^ Disease burden was estimated for each individual pulmonary artery segment and labeled on a scale of 0-4; zero (0) indicated no obstruction, one (1) indicated involvement of the distal segmental and sub-segmental branches, two (2) indicated proximal segmental artery involvement, three (3) indicated involvement of the lobar branches, and four (4) indicated proximal—main pulmonary artery disease. Disease burden was assessed by summing the assigned obstruction value (0-4) for each individual lung lobe.

Lung lobes were independently assessed through linear regressions. Disease burden values of 0 were excluded for the corresponding lobe on individual analysis. All lung lobes and the three (3) lobes with the highest *R*^2^ values when examining the relationship between disease burden and absolute change in PVR were summed to analyze combined disease burden effects on PVR.

### Statistical analysis

Continuous variables were reported using mean and standard deviation; categorical variables were reported as a frequency or percentage. PVR was obtained from both baseline and post-PTE RHCs. These values were utilized to calculate an absolute change in PVR. R^2^ was calculated to estimate the proportion of the absolute change in PVR that could be attributed to baseline obstruction location and severity before PTE.

## Results

### Study demographics

A total of 125 patients underwent PTE between January 1st, 2019, and March 31st, 2023, at the University of Michigan Hospital. Nine (9) patients were excluded due to pre-operative IPA not being performed in accordance with our center protocol, as IPA was completed without obtaining standard angiographic views. One (1) patient was excluded due to pre-operative PVR not being measured, and one (1) patient was excluded for having right main pulmonary artery total occlusion. Fifty-eight (58) patients were excluded as post-operative RHC was unavailable. Of these 58 patients, 36 were unavailable due to the patient care being returned to the referring institution, and 22 did not have follow-up RHC completed (five (5) were deceased, four (4) were not completed due to patient preference, four (4) were not completed due to patient frailty, and nine (9) were lost to follow-up). Therefore, our final cohort contained 56 patients, with 47 (83.9%) having World Health Organization (WHO)-FC III or IV symptoms. A history of acute pulmonary embolism was reported in 91.1% (*n* = 51) of patients. Fourteen patients had a medical condition that increased the risk of CTEPH, including thyroid disease (*n* = 5; 8.9%), thrombophilia (*n* = 6; 10.7%), antiphospholipid disease (*n* = 5; 8.9%), blood dyscrasia, anemia, or hemoglobinopathy (*n* = 7; 12.5%) and a history of a splenectomy (*n* = 1; 1.8%). Several patients also had other medical comorbidities, including atrial fibrillation (*n* = 6, 10.7%), chronic obstructive pulmonary disease/asthma (*n* = 16; 28.6%), and obstructive sleep apnea (*n* = 16; 28.6%) (Table 1).

**Table 1:** Baseline Demographics of Included Participants

Baseline characteristics	
Age	Mean: 57.2 ± 15.8 years old
Sex (Female)	*N* = 32 (60.0%)
Race	White: *N* = 42 (75.0%)
	Black: *N* = 13 (23.2%)
	Native Hawaiian and Other Pacific Islander: *N* = 1 (1.8%)
BMI	Mean: 31.9 ± 7.6 kg/m^2^
WHO-FC	II: *N* = 9 (16.1%)
	III: *N* = 42 (75.0%)
	IV: *N* = 5 (8.9%)
Other Pulmonary Hypertension Diagnoses	Left Heart-Disease Related: *N* = 2 (4.0%)
	Lung-Disease Related: *N* = 3 (5.4%)
History of DVT	*N* = 15 (26.8%)
Pre-Operative PVR > 1,000 dynes	*N* = 11 (19.6%)
History of Acute Pulmonary Embolism	*N* = 51 (91.1%)
History of Intravenous Device	*N* = 0 (0.0%)
Atrial Fibrillation	*N* = 6 (10.7%)
History of Thyroid Disease	*N* = 5 (8.9%)
History of Illicit Drug Use	*N* = 3 (5.4%)
History of Smoking	Current: *N* = 2 (3.6%)
	Prior: *N* = 19 (35.7%)
Coagulopathy	Thrombophilia: *N* = 6 (10.7%)
	Antiphospholipid Syndrome: *N* = 5 (8.9%)
Blood dyscrasia, anemia, or hemoglobinopathy	*N* = 7 (12.5%)
Post-Splenectomy	*N* = 1 (1.8%)
History of Malignancy*	*N* = 4 (7.1%)
History of Miscarriage	*N* = 4 (7.1%)
History of HIV	*N* = 0 (0.0%)
Cirrhosis and/or Portal Hypertension	*N* = 1 (1.8%)
COPD/Asthma	*N* = 16 (28.6%)
OSA	*N* = 16 (28.6%)

COPD, Chronic obstructive pulmonary disease; DVT, Deep vein thrombosis; HIV, Human immunodeficiency virus; OSA, Obstructive sleep apnea; PVR, Pulmonary vascular resistance; WHO-FC, WorldHealth Organization functional class.

* Excluding non-melanoma skin cancer.

A pulmonary hypertension specialist evaluated the patients in the inpatient or outpatient setting before PTE. In every included case, a well-defined protocol (previously published) was followed for the performance and interpretation of IPA before PTE.^8^ Pre-procedure RHCs were performed on average 85.5 ± 65.6 days before PTE. Post-PTE RHCs were completed on an average of 236.1 ± 126.3 days following PTE (Table 2).

**Table 2:** Operative Characteristics of Included Participants

Operative timing	
Time between initial RHC and PTE	85.5 ± 65.6 d
Time between PTE and Follow-Up RHC	236.1 ± 126.3 d
*Pre-operative*	
In ICU	*N* = 2 (3.6%)
On IV Inotropes	*N* = 0 (0.0%)
On Mechanical Ventilation	*N* = 0 (0.0%)
*Intraoperative*	
Total Surgical Time	402.5 ± 57.8 min
Total Cardiopulmonary Bypass Time	253.6 ± 41.8 min
Total Cross-Clamp Time	102.0 ± 30.2 min
Total Circulatory Arrest Time	56.5 ± 17.4 min
*Post-operative*	
Time Intubated	41.2 ± 69.1 h
Reintubated During Hospitalization	N = 4 (7.1%)
Post-operative Hospitalization Duration	14.3 ± 9.2 d
Post-operative ICU Duration	4.5 ± 7.8 d
*Complications*	
Right Heart Failure	*N* = 2 (3.6%)
Sepsis	*N* = 2 (3.6%)
Stroke	*N* = 0 (0.0%)
Pneumonia	*N* = 1 (1.8%)
Circulatory Shock	*N* = 8 (14.3%)
Pericardial Effusion	*N* = 9 (16.1%)
Tamponade	*N* = 1 (1.8%)
Pleural Effusion	*N* = 13 (23.2%)
Pulmonary Hemorrhage	*N* = 0 (0.0%)
Need for Intravascular Embolization	*N* = 0 (0.0%)
Need for Redo Sternotomy	*N* = 1 (1.8%)
Heart Block	*N* = 2 (3.6%)
Cardiac Arrest	*N* = 0 (0.0%)
Death	*N* = 1 (1.8%)

ICU, Intensive care unit; PTE, Pulmonary thromboendarterectomy; RHC, Rightheart catheterization.

Operative characteristics of the PTE procedure are outlined in Table 2. The mean cardiopulmonary bypass time was 253.6 ± 41.8 min, the mean cross-clamp time was 102.0 ± 30.2 min, and the mean circulatory arrest time was 56.5 ± 17.4 min. Postoperatively, the patients were monitored in the ICU for 4.5 ± 7.8 days before being transferred to the cardiac floor and had a mean total hospital stay of 14.3 ± 9.2 days. Patients remained intubated postoperatively for a mean time of 41.2 ± 69.1 hours, and four (7.1%) patients required reintubation during their hospitalization course. Twenty-four (24) patients suffered post-operative complications, including right heart failure (*n* = 2), sepsis (*n* = 2), pneumonia (*n* = 1), circulatory shock (*n* = 8), pericardial effusion (*n* = 9), tamponade (*n* = 1), pleural effusion (*n* = 13), need for repeat sternotomy (*n* = 1), and heart block (*n* = 2). During the same hospital admission, one (1) patient died 58 days post-PTE due to septic shock complicated by bowel ischemia but was included in this analysis as they underwent RHC 26 days post-PTE.

Pre-operative mean right atrial pressure was 14.9 ± 4.8 mmHg, which decreased to 8.9 ± 4.5 mmHg postoperatively. Pre-operative mean pulmonary artery pressure was 49.4 ± 12.0 mmHg, which decreased to 31.5 ± 11.1 mmHg postoperatively. Pre-operative thermodilution cardiac index (CI) was 2.1 ± 0.6 L/min/m^2^, which increased to 2.6 ± 0.6 L/min/m^2^ postoperatively. Finally, pre-operative PVR was 710.0 ± 343.1 dynes/sec/cm^5^, which decreased to 268.3 ± 181.4 dynes/sec/cm^5^ postoperatively.

### Modeling the effect of disease burden on hemodynamics

**Figure 1:**
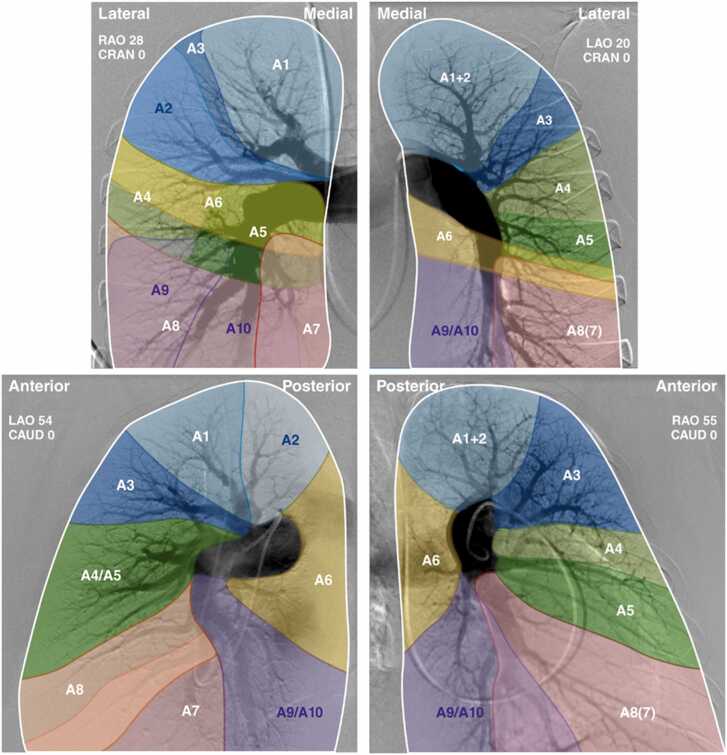
Lung perfusion zones with each bronchopulmonary segment labeled. In the right lung, upper lobe segments are A1, A2, and A3; middle lung segments are A4 and A5; lower lobe segments are A6, A7, A8, A9, and A10. In the left lung, upper lobe segments are A1, A2, and A3; lingular segments are A4 and A5; Lower lobe segments are A6, A8, A9, and A10. This figure is from the referenced AHA statement.^9^ AHA, American heart association.

**Figure 2:**
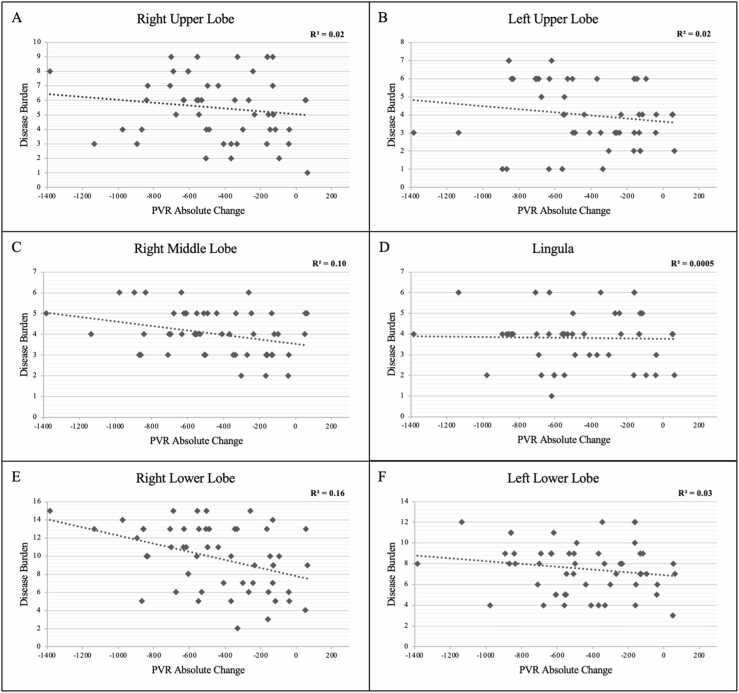
Relationship between absolute change in PVR and disease burden in each lung lobe. **(A)** RUL. **(B)** LUL. **(C)** RML. **(D)** Lingula. **(E)** RLL. **(F)** LLL.

**Figure 3:**
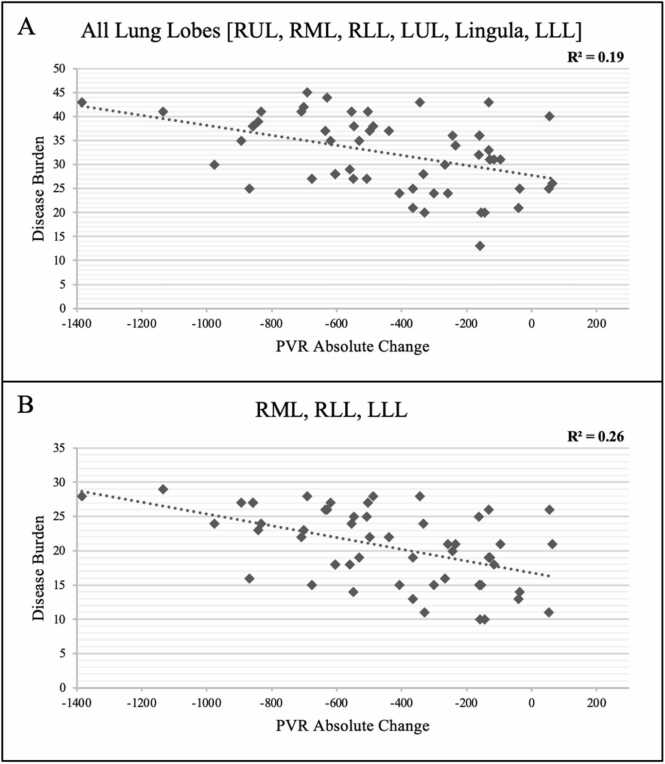
**(A)** Relationship between total lung disease burden and absolute change in PVR. **(B)** Relationship between disease burden involving the right middle (RML), right lower (RLL), and left lower (LLL) lobes and absolute change in PVR. PVR, pulmonary vascular resistance.

Absolute change in PVR was compared to the total disease burden calculated from the values assigned to each of the 19 bronchopulmonary segments, as shown in Figure 1. The cumulative disease burden for every patient was assessed by adding the disease burden score assigned to each segment. Disease burden in the right lower lobe (RLL) had the strongest correlation with hemodynamic response to PTE (*R*^2^ = 0.16), followed by right middle lobe (RML) (*R*^2^ = 0.10) and left lower lobe (LLL) (*R*^2^ = 0.03). Figure 2 shows correlation coefficients for hemodynamic response to PTE for each lobe of the right and left lung separately. Cumulatively, the aggregate disease burden of RLL, LLL, and RML was noted to have the strongest correlation with the hemodynamic response following PTE (*R*^2^ = 0.26) compared to the whole lung (*R*^2^ = 0.19, Figure 3).

## Discussion

The results of this study show a possible association between the anatomic location and laterality of the disease burden in CTEPH and hemodynamic response following PTE. While overall disease burden has been previously described to correlate with response to PTE, this study suggests that disease location and laterality, specifically in the RLL, RML, and LLL, in that order, may have an independent correlation with absolute change in PVR following PTE. This suggests that disease localized in these lobes may have a larger contribution to elevated pulmonary pressures compared to other vascular lung territories and may offer greater hemodynamic improvement following surgical revascularization.

It is important, however, to contextualize the strength of these associations. While the RLL, RML, and LLL showed the highest individual *R*^2^ values (0.16, 0.10, and 0.03, respectively), these values reflect only modest correlations. Even the highest-performing aggregate model incorporating RLL, RML, and LLL achieved an *R*^2^ of only 0.26. This implies that nearly three-quarters of the variability in post-PTE PVR change remains unexplained by disease location alone. This suggests that change in PVR post-PTE is likely multifactorial, related to other factors such as pulmonary vasculopathy burden and other patient-related factors.

Nonetheless, the observed trends, showing the most substantial correlation being in the RLL, RML, and LLL, are consistent with physiologic pulmonary blood flow, in which lower lung lobes are preferentially perfused for ventilation matching, regardless of supine or decubitus positioning or if a patient is exercising. While this is gravity-dependent primarily, normal pulmonary circulation is defined by lower resistance in these areas due to increased recruitment and distensibility of healthy vessels. When blood flow is shunted elsewhere, this will occur through a higher resistance environment due to gravity, distensibility, and vasoconstriction in poorer V/Q matching.10., 11 Therefore, restoring blood flow to these lower lung areas could be hypothesized to have the greatest impact in restoring a more normal PVR. Additionally, it is conceivable that the disease severity in the RML is also a marker of a higher disease burden in the RLL, which could be underappreciated on IPA due to proximal vessel sharing [interlobar artery]. This may also be a reason for RML disease being an important contributor to the hemodynamic response following PTE.

Previously, Renapurkar et al proposed a disease burden score using computed tomography and found that patients with higher disease burden experienced greater improvement after PTE. However, their analysis did not assess the differential impact of specific lung lobes on hemodynamic improvement.^5^ Though computed tomography pulmonary angiogram (CTPA) can be complementary in assessing disease burden in patients with CTEPH, CTPA lesion load assessment can further confound analysis, given its limitations in detecting disease at the level of the segmental and sub-segmental arteries.

While the modest *R*^2^ values in this analysis temper our conclusions, they also underscore an important opportunity to refine risk stratification by integrating anatomic, physiologic, and functional data into more comprehensive predictive models. Our findings introduce new knowledge regarding a differential contribution of various lung lobes towards hemodynamic derangement in CTEPH patients.

This study has several strengths. Firstly, using RHC to evaluate hemodynamic response after PTE enables a more precise assessment. Although this method excludes patients who did not survive the surgery, it allows for evaluating longer-term hemodynamic responses to PTE. Secondly, the disease burden was assessed on angiograms before surgical intervention, with the original IPA interpreter being blinded to patient outcomes. The results of this study should be taken in the context of several limitations, and we want to exercise caution with interpretation. Firstly, the cohort in question consisted of patients who underwent PTE. Thus, the decision-making and patient selection process for balloon pulmonary angioplasty should be distinct from these results. Moreover, all procedures were performed in a single PTE-capable center,^12^ and the sample size was small. Additionally, CTPA data was not utilized, though this could be considered complementary to IPA. Lastly, our analysis did not account for complete occlusions, as it primarily focused on disease location rather than disease subtype (focal lesions, web-like lesions, subtotal occlusions, total occlusions, etc).

## Conclusion

These findings underscore the importance of disease location in the lower lung lobes and the RML as important determinants for hemodynamic response to PTE in patients with CTEPH. This is particularly relevant in the current era, given the alternative of minimally invasive balloon pulmonary angioplasty, which has been shown to be safe and efficacious,^13^ for pulmonary arterial revascularization in patients with CTEPH.

## Author Contributions

V. Aggarwal conceived the study. B.O. Pérez Martínez, G.V. Rubick, and A. Toiv collected data and performed analysis. The remaining authors contributed additional data and approved the study manuscript.

## Disclosure Statement

V.V. McLaughlin reports grants from PI-Aerovate, Enzyvant/Altavant, Gossamer-Bio, Keros, Sonovie, Sub-I Janssen, Merck/Aceleron, and consulting fees from Aerami, Aerovate, Altavant (ended within 1 year), Apollo, Bayer, CVS/Caremark, L.L.C., Corvista, Gossamer Bio, Janssen, Keros, Liquidia, Merck, Morphic, Regeneron, Respira, Roivant, United Therapeutics, and Vertex. T.M. Casciono reports grants from Johnson & Johnson and consulting fees from Merck. The remaining authors have no potential conflicts of interest to disclose.

## Declaration of Competing Interest

The authors declare that they have no known competing financial interests or personal relationships that could have appeared to influence the work reported in this paper.

## Financial Support

No funding support was utilized for this study/research.
